# Study on an Indoor Positioning System for Harsh Environments Based on Wi-Fi and Bluetooth Low Energy

**DOI:** 10.3390/s17061299

**Published:** 2017-06-06

**Authors:** Gabriel de Blasio, Alexis Quesada-Arencibia, Carmelo R. García, Jezabel Miriam Molina-Gil, Cándido Caballero-Gil

**Affiliations:** 1 Instituto Universitario de Ciencias y Tecnologías Cibernéticas, ULPGC, 35001 Las Palmas de Gran Canaria, Las Palmas, Spain; aquesada@dis.ulpgc.es (A.Q.-A.); rgarcia@dis.ulpgc.es (C.R.G.); 2 Department of Computer Engineering and Systems, University of La Laguna, 38200 San Cristóbal de La Laguna, Santa Cruz de Tenerife, Spain; jmmolina@ull.es (J.M.M.-G.); ccabgil@ull.es (C.C.-G.)

**Keywords:** indoor positioning system, ubiquitous computing, intelligent transport system, WLAN, BLE

## Abstract

This paper presents a study of positioning system that provides advanced information services based on Wi-Fi and Bluetooth Low Energy (BLE) technologies. It uses Wi-Fi for rough positioning and BLE for fine positioning. It is designed for use in public transportation system stations and terminals where the conditions are “hostile” or unfavourable due to signal noise produced by the continuous movement of passengers and buses, data collection conducted in the constant presence thereof, multipath fading, non-line of sight (NLOS) conditions, the fact that part of the wireless communication infrastructure has already been deployed and positioned in a way that may not be optimal for positioning purposes, variable humidity conditions, etc. The ultimate goal is to provide a service that may be used to assist people with special needs. We present experimental results based on scene analysis; the main distance metric used was the Euclidean distance but the Mahalanobis distance was also used in one case. The algorithm employed to compare fingerprints was the weighted *k*-nearest neighbor one. For Wi-Fi, with only three visible access points, accuracy ranged from 3.94 to 4.82 m, and precision from 5.21 to 7.0 m 90% of the time. With respect to BLE, with a low beacon density (1 beacon per 45.7 m^2^), accuracy ranged from 1.47 to 2.15 m, and precision from 1.81 to 3.58 m 90% of the time. Taking into account the fact that this system is designed to work in real situations in a scenario with high environmental fluctuations, and comparing the results with others obtained in laboratory scenarios, our results are promising and demonstrate that the system would be able to position users with these reasonable values of accuracy and precision.

## 1. Introduction

In modern societies the mobility of citizens is considered to be both a necessity and a right. Consequently, transport systems play a fundamental role in the economic and social development of these societies. The widespread use of transport systems, especially road transport, has resulted in problems related to pollution, safety and environmental degradation. All transport planning bodies agree that public transport systems need to be developed in order to minimise these problems. The key aspects for making public transport more attractive than private transport are accessibility and safety. In the case of people with special needs, these aspects are even more important—if that is possible—since particular attention is required when developing public transport network access that meets their needs. According to the United Nations, 15% of the world’s population has some kind of disability [1]. Therefore, the development of accessible public transportation infrastructure is not only a technological challenge, but it is also an ethical obligation if more inclusive and fairer societies are to be developed. For this reason, transport authorities have issued recommendations and regulations that address this problem; for example, the European Union considers this to be an aspect that should be taken into account when developing the Smart City paradigm [2]. According to Mitchell et al. [3], the situations in which a person with special needs requires special attention when using public road transport are: when gaining access to and using the services of stops and stations, boarding and alighting from the vehicle, and making payment for the trip.

**Figure 1:**
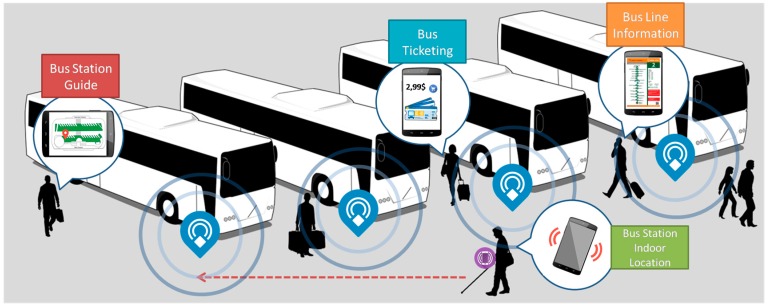
Schematic view of advanced information services that could be offered: bus station guide, ticketing, line information.

The challenge addressed by this article is positioning in public indoor spaces, where the environmental conditions that affect the propagation of the signal used to determine the location are very variable and may therefore affect accuracy and precision in relation to the actual location. This article focuses specifically on indoor spaces on the public transportation network, in which these conditions vary according to the time, day or time of year. Another facet of the challenge is to achieve suitable accuracy and precision in the positioning by making use of the wireless communication infrastructures that already exist in these places of the transport network. Since these elements had previously been installed for purposes other than those of our study, they were not optimally configured for positioning. The ultimate aim is to provide a service to assist people with special needs, such as people with visual impairments or cognitive problems, who may feel disoriented in public spaces that are new to them. The proposed system obtains the user’s position through the combined use of a wireless local area network (WLAN) and Bluetooth Low Energy (BLE) at the station used for the study. In addition to indoor positioning, BLE beacons are especially interesting for stations of public transportation systems because they can be used for other advanced information services such as bus station guides, ticketing or line information (Figure 1).

The main contributions of this system are: first, it provides a real implementation of a positioning system in an environment that is not favourable to the use of these technologies; second, it does not require any additional deployment of the available WLAN infrastructure, using the elements already installed in the bus stations; and, third, it studies the behaviour of different positioning methods, proposed by different authors, using these technologies to monitor their behaviour in environments with changing conditions (variations in the number of people in the station according to the time of day, variations in environmental conditions, such as humidity, and the location of the Wi-Fi access points (AP) and Bluetooth beacons at points that are not optimal for positioning purposes).

This article is divided into seven sections. In the following section we will describe the studies relevant to the proposed system. In the third section we review the most important indoor positioning techniques and describe the testbed where we performed the different tests. The proposed positioning system is presented in the fourth section. In Section 5 the different tests that were carried out using the Wi-Fi and BLE infrastructure are described and analysed, showing in each case the results that are obtained. In Section 6, a comparison of our results with results from other studies is presented. Finally, the conclusions and future lines of work are presented in the seventh section.

## 2. Related Works

In this section we analyse various studies of positioning in indoor spaces. This list of works has been organised into five groups. The first consists of studies that use Wi-Fi technology for positioning. The second group consists of studies that use Bluetooth technology for positioning. The third group comprises studies on the combined use of these two technologies for positioning. Proposals for positioning systems designed to assist people with special needs are covered in the next group. Lastly, in the fifth group we review studies that address positioning in large public spaces. 

In the context of indoor positioning systems that use Wi-Fi technology, it is worth noting the pioneering work of Bahl et al. [4], who proposed the RADAR system. It is also worth mentioning the work of Liu et al. [5], which offers a broad overview of existing wireless indoor positioning solutions and attempts to classify different techniques and systems. Honkavirta et al. [6] outlined positioning methods that use scene analysis, as did He et al. [7], although the latter focused on more recent developments. Kaemarungsi et al. [8] conducted an interesting and exhaustive study of the statistical properties of received signal strength (RSS) for position location by scene analysis. In the work of Dawes et al. [9], deterministic and probabilistic indoor positioning methods were compared on the same testbed. In relation to Wi-Fi fingerprinting, Torres-Sospedra et al. [10] observed that most studies use Euclidean distance and raw data, and therefore carried out a study of the best distance function, the best way to represent the data, and the effect of applying thresholding techniques. Other studies we may mention include that by Feng et al. [11] which, among other contributions, refined the search of an RSS fingerprint database by clustering using an affinity propagation algorithm. Kjærgaard et al. [12] studied the problem of location fingerprinting with heterogeneous wireless clients. King et al. [13] presented a detailed analysis of the deployment, calibration, and measurement factors that cause positioning errors.

The use of Bluetooth technology and, more specifically, Bluetooth Low Energy technology has been proposed by several authors. Faragher et al., in two important papers [14,15], first investigated the impact of BLE devices on indoor positioning schemes based on RSS fingerprints, and then conducted a detailed scene analysis study using proximity and the *k*-nearest neighbours (KNN) algorithm with the aggregation of the three BLE channels. Zhuang et al. [16] used an algorithm that combines a polynomial regression model, fingerprinting with channel separation, outlier detection, and Kalman filtering. Kajioka et al. [17] demonstrated the viability of positioning through the received signal strength of BLE beacons.

Some authors have proposed the use of hybrid systems that combine Wi-Fi and Bluetooth technology in order to overcome their limitations for positioning purposes. Baniukevic et al. [18] developed an algorithm that prevents positioning errors caused by reference positions that are similar by separating these positions into different smaller radio maps through the deployment of beacons at particular locations. Metola-Moreno et al. [19] compared two different positioning algorithms using Bluetooth and WLAN: the first is based on the construction of a fusion map using the Wi-Fi and Bluetooth RSS values; in the second algorithm, the position is determined independently by each technology and the results are subsequently combined.

A particularly notable application of indoor positioning systems is the provision of assistance for people with special needs. Au et al. [20] proposed an indoor tracking and navigation system based on RSS measurements in a wireless local area network (WLAN). The location determination problem was solved by first applying a proximity constraint to limit the distance between a coarse estimate of the current position and a previous estimate. Then, a compressive sensing-based positioning scheme was applied to obtain a refined position estimate. Moder et al. [21] focused on the abilities of an indoor positioning system purely based on sensors that are already present in smartphones. Algorithms were designed to process the accelerometer, gyroscope, magnetometer and barometer data, and Wi-Fi fingerprinting; the results were then passed through a mathematical filter to obtain a final position and heading information. In the case of Bluetooth-based systems, Ge [22] implemented two indoor positioning systems and a specific interface. The first system uses beacon selection and pseudo-intersection for pre-processing, and triangulation and fingerprinting as the main algorithms. It achieves a positioning accuracy of 1.83 m. The second system uses a proximity algorithm, and a specific user interface was tailor-made for blind and visually impaired users of these two systems. Guerrero et al. [23] presented an indoor navigation system that identifies the position of a visually impaired person and calculates the velocity and direction of their movements. Castillo-Cara et al. [24] presented a prototype of an indoor mobility assistant for visually impaired users. The system uses the RSS provided by BLE beacons strategically placed to identify different areas of a building, using also the pedometer and gyroscope of a smartphone.

The final area of related studies concerns positioning system proposals for indoor public spaces on public transport networks. In such indoor environments—bus, metro or train stations, shopping centres, etc.—there may be a lot of noise produced by the presence of many people or by humidity. We may mention the study conducted by Ladd et al. [25], in which the system design begins with the observation that the determination of position from complex, noisy and non-Gaussian signals is a well-studied problem in the field of robotics; a robust position estimation to within a metre is achieved in an experimental context. Lin et al. [26] introduced an enhanced indoor location algorithm based on the Redpin algorithm, which matches the received Wi-Fi signal with the signals in the training data and uses the position of the closest training data as the user’s current location. Dickinson et al. [27] introduced a framework for the positioning of users in a large wholesale shopping store, presenting results obtained using different methods of positioning and using RSS measurements. Insoft GmbH [28] has created an app for Swiss Federal Railways (SBB), which helps passengers to find their way through Zurich’s multilevel main station, which occupies a space of approximately 175,000 m^2^. The app uses more than 1000 Bluetooth beacons and sensor fusion to locate the position of the smartphone.

## 3. Preliminaries

### 3.1. Positioning Techniques

Indoor positioning in public spaces on the transport network is a challenge for several reasons: during propagation, the radio signal is subject to reflection, diffraction and scattering due to the architectural elements that exist in that space. To these effects we also have to add signal attenuation due to the presence of people, which may be a mass presence of people in public spaces such as public transport stations, airports, shopping centres, etc. The signal is also affected by other factors, including ambient humidity conditions [7,8].

One of the most-used indoor positioning techniques are wireless local area networks (WLANs) is scene analysis or fingerprinting, a process by which radio signals are measured and associated with positions. A position is then characterised by the signal pattern detected from each Wi-Fi AP [7]. For systems deployed in large spaces, in order to estimate the position of a mobile device user, it is necessary to previously construct (offline phase) a location fingerprint database or correlation database (CDB) for a set of reference points of known positions [4,29]. This database is also known as a radio map, in which each reference element or fingerprint consists of the coordinates of the reference point, the received signal strength (RSS) of each AP, the orientation in which these RSS readings have been taken, etc. Each element of the CBD is a mapping between the position and the distribution of the RSS values [8]. Subsequently (online phase), users in an unknown position initially obtain the RSS values for the different APs (target fingerprint) with their mobile device and, by means of some method, they compare these RSS values with those stored in the database, to ultimately obtain the coordinates of their location. The position of the user may be determined primarily through deterministic [4] or probabilistic [30] algorithms.

For deterministic algorithms, similarity metrics are used to compare the fingerprints stored in the offline phase with the measurements taken in the online phase: the user is located at the coordinates for which the reference fingerprint is at the minimum distance in the signal space of the target fingerprint. Many distance metrics can be used (e.g., Euclidean, Mahalanobis, Manhattan, etc.) but the user’s position is obtained, irrespective of the metric used, through the coordinates associated with a reference fingerprint stored in the database [29]. The comparison of fingerprints using deterministic methods may be performed using KNN or some other pattern-matching algorithm, such as those used in artificial neural networks or support vector machines.

Probabilistic algorithms are based on statistical inference. For this type of algorithm, a set of training data is used that searches for the position of the user with the maximum likelihood [30]. In this paper we will focus on deterministic methods. The main advantages and disadvantages of indoor scenario analysis are [7]:

1. Ease of implementation, using an existing infrastructure without the need to introduce new hardware, making it a low-cost option for which RSS data are easily obtained, computational complexity is low, and reasonable accuracy and precision are achieved.
2. Much time must be devoted to constructing the radio map or database, changes in hardware or in the distribution of furniture can make the stored fingerprints obsolete, thus affecting the accuracy and precision of positioning, the performance of these methods depends to a large extent on the practical implementation parameters and on the nature of the environments in which they are implemented, RSS values can be affected by the different types of devices used to create the database and by mobile users.

### 3.2. Deployment Environment

**Figure 2:**
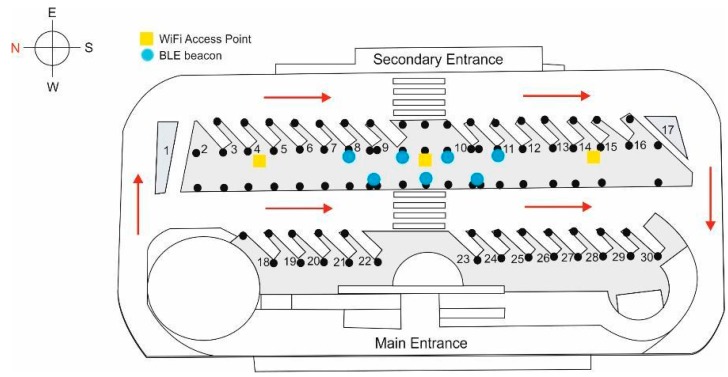
Schematic view of key elements of the bus station and Wi-Fi APs and BLE beacons.

The scenario in which the positioning system will be tested is a local bus station. The schematic diagram of the station in Figure 2 shows the most important elements: the two boarding areas (areas of interest East (E) and West (W)), which are shaded in grey, the distribution of the Wi-Fi APs and the BLE beacons (in area of interest E), bus paths (red lines) and stops (numbered), columns (black circles) and, finally, pedestrian crossings.

**Figure 3:**
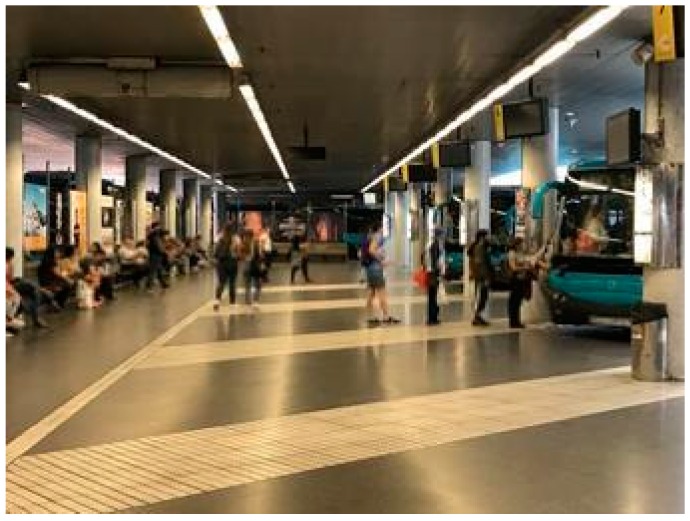
View of the area to be used as a testbed.

The bus station is built of reinforced concrete, with concrete columns approximately every 6 m on a north-south axis, and every 8 m on an east-west axis. There is a suspended ceiling at a height of 3.76 m, where the Wi-Fi access points are located. On one side of the corridor in the area of interest, and adjoining the seating area of benches where users may wait for the different bus lines to depart (on the left side of the photo in Figure 3), there is a glass window (except in the central section) with a thickness of 1 cm and a height of approximately 2.5 m (Figure 3). It should be noted that the station is located very close to the sea, so ambient humidity is a factor to consider

Users access the station by two entrances (main and secondary) and then move to areas of interest, either directly (no stairs or ramps) or using disabled access ramps or stairs. Note that when entering the station the system must locate users in one of the two zones of interest and then provide them with a service (information about a bus line, etc.).

From the above scenario, and taking into account the fact that the station’s three Wi-Fi access points have already been deployed and that, although others from the outside are detected, their signal is very weak and unreliable, it is necessary to deploy BLE beacons to meet two objectives:

1. The signals reach all areas with sufficient intensity, particularly areas of interest.
2. Positioning in those areas is correct, there are no positioning errors that will lead users to danger zones, and the user is provided with the desired advanced services.

The chosen model meets objectives (1) and (2), but gives special consideration to the safety and convenience of users. Due to the particular distribution of architectural elements in the bus station (columns, etc.) and to prevent positioning errors on mobile devices, a strategy was implemented to deploy BLE beacons near the bus stops with the aim of also providing users with advanced services.

Because the Wi-Fi APs and BLE beacons share the same frequency range (2.4 GHz) there may be interference problems [15], although: (1) interference problems usually occur when the number of access points is very large, something that did not happen in our case; (2) possible interference problems could be overcome by avoiding certain channels and taking into account the fact that Bluetooth automatically uses FHSS (frequency hopping spread spectrum).

**Figure 4:**
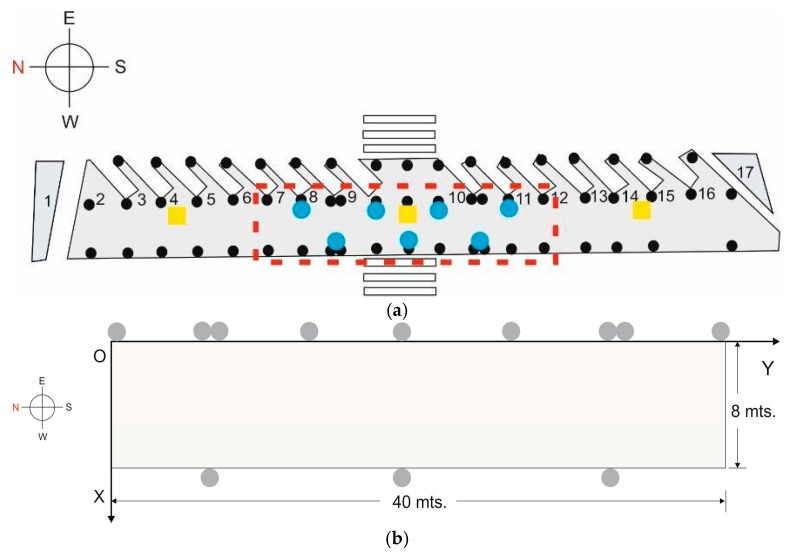
Testbed: (**a**) schematic view (highlighted in red); (**b**) dimensions and axes of chosen coordinates.

Within the bus station described above, a central section of one of the areas of interest was chosen as the testbed. This is possibly the zone of the station with the greatest difficulty for positioning due to the continuous transit of users and of buses in the surrounding areas. Within the area of interest, we selected a 40 m × 8 m rectangular section. Figure 4 shows the section (marked with a red rectangle) and the chosen origin of coordinates, O, as the reference for the coordinates of the points in the database.

**Figure 5:**
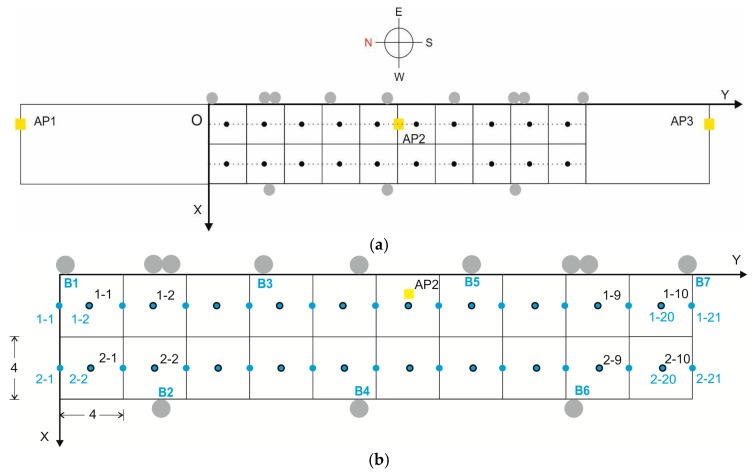
Grid chosen in the testbed: (**a**) in relation to the 3 Wi-Fi APs; (**b**) detail showing the reference points of the Wi-Fi and BLE databases and the arrangement of the BLE beacons.

To create the database of Wi-Fi reference points, and considering that the accuracy that we will require from this positioning system is low, we chose a grid of 20 cells, each measuring 4 m × 4 m (Figure 5a), with the reference points aligned on two parallel lines (which we have labelled as lines 1 and 2), and each reference point in the centre of each cell [13]. Line 1 is closer to the passenger access to the buses, while line 2 is further away, near the window and a seating area. The testbed therefore has an area of 320 m^2^, and consequently a density of 16 fingerprints per m^2^ [9]. The brand of the Wi-Fi access points that were already deployed in the station is Ubiquiti Networks, model UniFi 802.11ac Long Range Access Point, with a maximum TX power of +20dBm. And their coordinates with respect to O are AP1 = (2.2, −19.1), AP2 = (2.2, −20.4), AP3 = (2.2, 53.9), which means that they are aligned along reference points line 1 (see Figure 5a) and practically in line of sight (LOS) conditions. We noted that AP1 used channel 13, and AP3 used channel 11 so, even though there is a distance of about 73 m between them, this could potentially be a source of interference between the two APs and may add more noise to the system. AP2 used channel 1, so it would not be a source of interference.

To construct the BLE database, a structure similar to that described above was chosen, but taking into account the fact that the accuracy required for the BLE positioning system is greater: we therefore chose initially a grid of 22 cells, each measuring 2 m × 2 m with a density of 14.5 fingerprints per m^2^ (Figure 5b). The number of reference points was increased later to 42 cells. The seven deployed beacons are model iBKS105 units (Accent Systems, Barcelona, Spain), with a maximum output of +4 dBm. It should be noted that on both grids (Wi-Fi and BLE) there are common reference points.

It is important to note that the signal from the three Wi-Fi access points installed by the local transport authorities is detected with sufficient power within the station, but due to the concrete structure of the station, the RSS of other Wi-Fi access points on the outside are too weak and practically non-existent for the majority of data readings. For this reason inclusion of these access points in the database unfortunately had to be ruled out.

## 4. Proposed Positioning System

In this section a new multilayer two-channel system to help indoor positioning is described. First we describe a generic location mechanism with a lower level of accuracy that roughly provides information on the existing environment and location (Wi-Fi channel). This will be a first mental map of what may be found in the environment in which the user wants to move. Then we describe a more accurate positioning system that provides more detailed environment information and allows us to locate and reach the exact site (BLE channel). This second channel, in addition to assisting the user in determining their location, also gives access to advanced information services (schedule information, etc.) using the first channel. We therefore propose a multilayer two-channel system that provides an initial level of abstraction that is subsequently refined to allow the user to reach their desired target. The system abstraction levels have been created to resemble the spatial perception of human beings as closely as possible.

### 4.1. Precision Levels: Definition of Zones

This first level of abstraction was created to provide knowledge or first awareness of the environment and surroundings. The aim is to provide users with some awareness of their location in the surrounding space, the environment and the objects in it. 

Having good spatial perception allows users to determine their location, move in this space, get their bearings, head in multiple directions, analyse situations and represent them. The zones are a key concept in this proposal. When people are confronted with a new environment, it is very useful for them to have a generic view of what is around, and then to specify the information required to reach the target point. For example, when approaching a station, in many cases it makes no sense to give a person instructions without first providing information on the surrounding environment. Therefore, in this proposal, two levels of accuracy are defined, depending on how close or how far users are from their goal. In particular, different parts of the protocol run depending on the zone where the user is in relation to their final goal.

**Figure 6:**
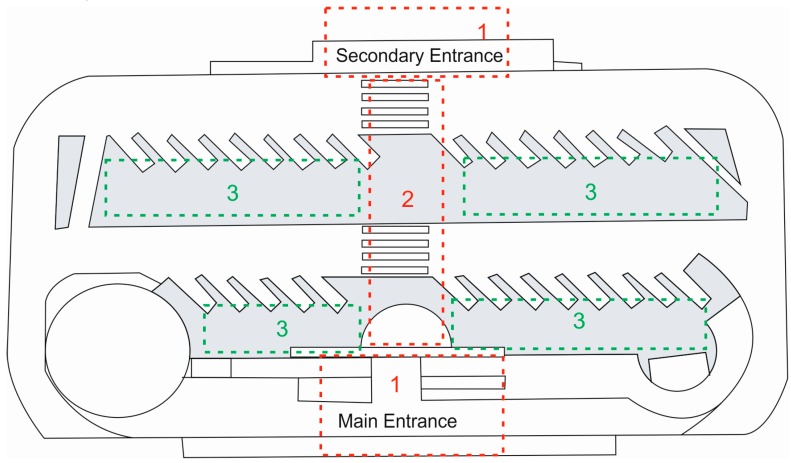
Schematic view of abstraction zones in a real scenario.

As shown in Figure 6, three zones are defined in a real scenario (schematic view of a local bus station), depending on the user’s location relative to their ultimate goal:

- First abstraction level: in this zone, users do not have direct contact with their target so they will be provided with a generic visualisation of the environment, emphasising the location of their target.
- Second abstraction level: users are not yet in the destination zone but receive information from both their target and the environment.
- Third abstraction level: users are in their target location where the target signal may be detected directly.

The size of the radius within which the different technologies come into play will depend on the environment where the users are using the system, taking into account factors such as coverage, complexity of the environment in which they move, distance to the target, etc. In Section 5 a use case is proposed in which the proposed system is deployed in a bus station.

### 4.2. Positioning System

When a user arrives, in this case, at a bus station, the system has to define their ultimate goal. At the first level of abstraction, Wi-Fi technology will come into play, which, depending on the user’s location and with respect to the Wi-Fi Access Points, will prompt the user as to the rough distribution of their environment. For example, “stairs to the right”, “cafeteria 50 m left”, “platforms 30 m ahead”, etc. Thus, users can make a mental map of where they are and where everything in their environment is, and the system will provide a first level of information that may be useful for a user who has never been to the station before. Otherwise, if the user has been to the station before, he or she will know where they are by comparison to previous occasions. At this time, and assuming that the user is using an application on their mobile phone, this will indicate where he or she must go, immediately receiving the first instructions. 

Obviously this first positioning system, as discussed above, works relatively well when little precision is required, since it is affected, on the one hand, by the wide range of sensors that current devices handle, because the received signal strength depends on them; and, on the other hand, by the number of people who are in the place, since up to 60% of the human adult body is water, and water absorbs radiation, so the signal will be affected [31]. Therefore, and as already mentioned, this single system does not solve the problem. Once users receive a first approximation, they will start walking, following the initial instructions. As they move, they will not only receive Wi-Fi signals but also BLE signals, so their route will be refined. Once the second level of abstraction is reached, the BLE devices, along with the Wi-Fi APs, will provide more information, reducing the limitations of each separate technology and complementing each other. 

However, there will come a point where all the signals overlap and it will be almost impossible to determine where the exact spot is (in this case, the bus platform that the user needs to locate). In this case it will be necessary to reduce the intensity of the BLE signal so that the user can only receive it when they are exactly where they expect to be. This can be achieved by configuring the transmission power of the beacons.

## 5. Performance Evaluation

For all the tests (both Wi-Fi and BLE) and owing to its simplicity and acceptable results, the distance metric used was the Euclidean distance—Equation (1)—except in one case in which Mahalanobis distance was used—Equation (2). The algorithm employed to compare the fingerprints was *k*-nearest neighbours, with a weighting inversely proportional to the distance (weighted *k*-nearest neighbours, WKNN)—Equation (3):

(1) $$d_{E}=\sqrt{\overset{N}{\underset{i=1}{\sum }}{\left(t_{i}-r_{i}\right)}^{T}\cdot \left(t_{i}-r_{i}\right)}$$

(2) $$d_{M}=\sqrt{\overset{N}{\underset{i=1}{\sum }}{\left(t_{i}-r_{i}\right)}^{T}\cdot \Sigma^{-1}\cdot \left(t_{i}-r_{i}\right)}$$

(3) $$\left(x,y\right)=\frac{\sum_{i=1}^{k}\left(x_{i},y_{i}\right)\cdot w_{i}}{\sum_{i=1}^{k}w_{i}}\;,\;\;\;\;\;\;w_{i}=\;\frac{1}{d_{i}}$$

where $t_{i},\;r_{i}$ are the target and reference RSSs respectively for access point i , Σ is the covariance matrix, (x,y) , $\left(x_{i},y_{i}\right)$ are the estimated coordinates of the target point and the coordinates of the k reference points and $w_{i}$ are the weights for each distance $d_{i}$ . For this study, the positioning accuracy is expressed by the mean error and its precision by the cumulative probability function (CDF), which is expressed in practice in percentile format [5].

### 5.1. Wi-Fi Analysis

#### 5.1.1. Preliminary Analysis

Before carrying out a Wi-Fi test, different analyses were carried out over several days to study the properties of the RSS in the testbed and in changing conditions of presence and movement of passengers and buses, the suitability of the data-measuring devices, etc.

On one day a 9-h test was conducted with two different laptops: an Asus N56J with a Ralink integrated wireless network card (Ralink Technology Corp., Taiwan) and an Acer Aspire 5750G with a Broadcom wireless network card (Broadcom Ltd., Irvine, CA, USA). Measurements were taken with the laptops on tables fitted with wheels and located at a height similar to that of a mobile carried in someone’s hand.

**Figure 7:**
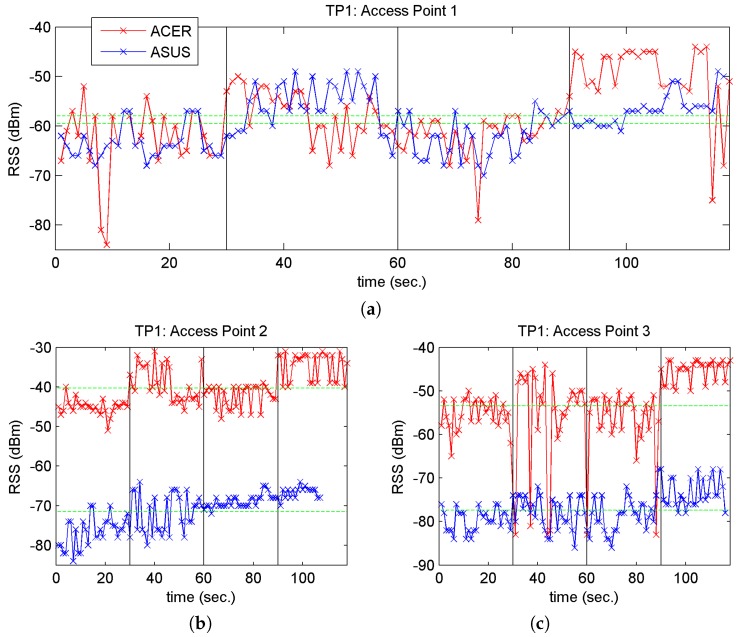
RSS vs. time for point TP1 and the three APs. (**a**) AP1; (**b**) AP2 and (**c**) AP3.

In the first test, samples were taken every 2 s for 1 min at different points and for each orientation (i.e., compass direction) (120 samples); the two laptops were used to take readings at different points on line 1 (closest to the passenger boarding area). The data were first recorded with one laptop and immediately after with the other. Figure 7 shows the time-related RSS values for the 3 APs and for one point, TP1; we labelled the points TP1 to TP4, with coordinates TP1 = (2.00, −32.45), TP2 = (2.00, −19.03), TP3 = (2.00, −6.83), TP4 = (2.00, 12.69), and which correspond to physical points in the vicinity of AP1 = (2.20, −19.1).

**Figure 8:**
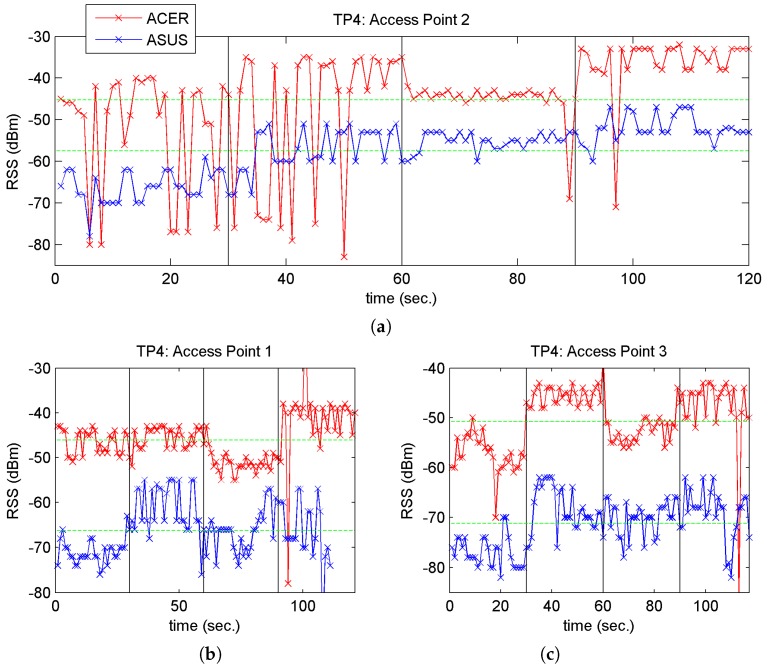
RSS vs. time for point TP4 and the three APs: (**a**) AP2; (**b**) AP1 and (**c**) AP3.

Both Figure 7 and Figure 8 represent change of orientation with vertical lines and the mean RSS values recorded by each laptop with green horizontal lines. When we study these figures closely, we observe the following:

(a) A change in the RSS when changing the orientation of the laptop, perhaps more pronounced in the Acer laptop: an increase in the RSS value was detected for E and W (between the 30–60 s time interval and the 90–120 s interval). This may be due to the combination of two causes: (1) that the APs are arranged on a north-south axis and the person taking the measurements partially screens the RSS from the APs (to a greater or lesser extent depending on the relative position of the measuring point and the position of the AP); and (2) the position of the antenna inside the laptop.
(b) The mean RSS values for the two laptops at TP1 are very close for AP1 (and more distant for AP2 and AP3), bearing in mind that TP1 is the closest point to AP1. This may be due to the large-scale-fading component that describes signal attenuation, since in some cases the signal travels more or less distance depending on the AP, and is more or less absorbed by people and materials on its path to the receiver. This component predicts the mean RSS value [8].
(c) The fluctuations in the RSS are greater for the Acer laptop than the Asus. This may be due to the increased movement of people and buses during the period of measurement with this laptop (although it was observed in almost all cases) or perhaps to the greater sensitivity of this laptop’s Wi-Fi card.

The observations in Figure 8a,c were also true for the other TP points. In relation to the phenomenon described in Figure 8b, the same occurred with the other TP points and their corresponding nearest access point. For example, in the case of point TP4, as it is located near AP2, the mean RSS values are very close, as may see in Figure 8.

These findings led us to use only the Asus laptop in subsequent positioning tests but with the possibility of conducting a more in-depth study for offline device calibration [9] in the future.

**Figure 9:**
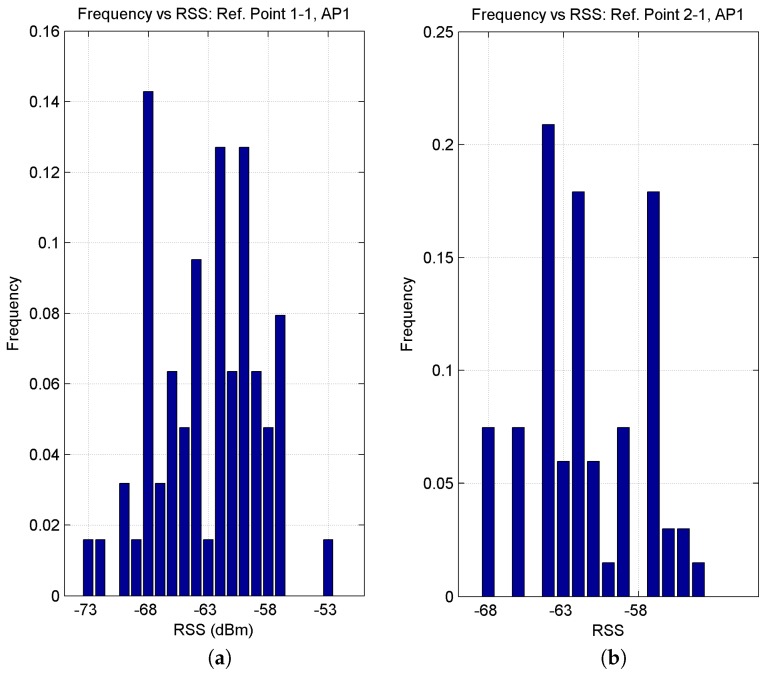
RSS histograms for AP1 at reference points (**a**) 1-1 and (**b**) 2-1.

The influence of people and buses and the influence of the position of the reference points were also analysed, i.e., their proximity to or distance from the passenger boarding area (near line 1 or line 2). For example, and for reasons of space, we shall only mention the AP1 RSS readings taken by the Asus laptop at two points: samples taken at reference points 1-1 (closer to the boarding area and at a distance of 19.03 m from AP1) and 2-1 (further from the boarding area and at a distance of 19.95 m from AP1), taking into account the fact that both points are separated by 6 m. As may be seen in Figure 9, the histogram for point 1-1 shows a wider RSS value range in comparison to the lower range shown in the histogram for point 2-1. The greater accumulation of people (queues) and their movements in the boarding area in relation to the seating/window area (and whether the data were measured in rush hour or not), would explain the behaviour of the RSS in these histograms.

The geometric arrangement of the 3 access points, mentioned in Section 3.2, led us to study its possible influence on the Wi-Fi positioning results: within the testbed and under LOS conditions we calculated the three straight lines parallel to the X axis of points at an equal distance between 2 pairs of access points. This was to enable us to determine the areas within the testbed with the highest RSS values.

**Figure 10:**
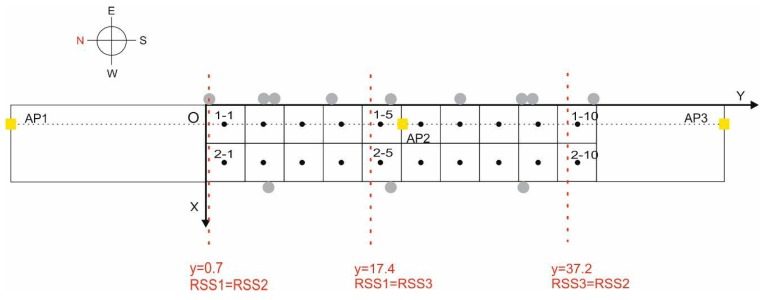
Straight lines with equal RSS values for Wi-Fi access point pairs.

Figure 10 shows the result of this calculation. Due to the relative configuration between the testbed and the AP distribution, it is interesting to note that AP2 has the greatest influence on most cells of the grid, i.e., theoretically and under LOS conditions, the order of the mean RSS values in the grids should be as follows:

- Cells 1 to 4: RSS2 > RSS1 > RSS3
- Cells 5 to 9: RSS2 > RSS3 > RSS1
- Cell 10: RSS3 > RSS2 > RSS1

This would imply that, theoretically at least, 3 zones would be created in the testbed in which the RSS order would be as indicated above. Table 1 shows data relating to one of the tests carried out in which this order is practically achieved, except for cells 1-1 and 2-3. These findings would serve for a future study in which the reference points could be grouped by some pattern-recognition procedure, e.g., propagation affinity, thus filtering and speeding up a search in a densely populated database of reference points [11].

#### 5.1.2. Empirical Approach to the Wi-Fi Tests

The study conducted by [13] provided a set of recommendations for a range of significant parameters to consider when gathering data:

(1) The number of APs is a primary factor in position errors.
(2) For the offline phase, 20 samples at each reference point is sufficient.
(3) For the online phase there is no ideal value for the number of samples to be used: a balance needs to be struck between improving the position error and the time needed to calculate the position. The study recommends using three samples although it points out that using 15 samples gives a reasonable cost-performance ratio.
(4) The ideal spacing between cells is 0.5 m but, on the other hand, it would take a lot of time to collect the data: the authors recommend using a spacing of between 1 and 2.5 m. With favourable values for these parameters, a minimum mean error value of 2.0 m is observed.

With these recommendations in mind, and considering our goal of “rough” Wi-Fi positioning, in the offline phase, with an ASUS N56J laptop with an integrated Ralink wireless network card, and Wi-Fi scanner software (Vistumbler), 30 one-second samples were taken at each reference point every 2 s (15 samples) for each access point and each orientation (N, E, S, W). By means of a laser pointer with an optical viewfinder with a range of up to 200 m and a precision of 1.0 mm, and for each reference point, the coordinates (*x*, *y*) were taken with respect to the coordinate source, O, of the grid. Therefore, each entry in the database has the generic structure $\left(xr_{i},yr_{i},R_{i}\right)$ in which $\left(xr_{i},yr_{i}\right)$ are the coordinates of each reference point i , and $R_{i}$ has the following structure:

(4) $$R_{i}=\left[\begin{array}{ccc} id_{j} & or_{k} & rss_{jk}\\ \vdots & \vdots & \vdots \end{array}\right]$$

where the index i=1−1,…,2−10 indicates the data collection line and the reference point, $id_{j}$ refers to the Wi-Fi access point with j=1,2,3 , $or_{k}$ refers to the orientation with k=N,E,S,W , and finally, $rss_{jk}$ indicates the received signal strength from access point *j* and with orientation *k*. Therefore, the database of reference points consists of approximately 3600 entries (20 points × 15 samples × 4 orientations × 3 access points). Once the raw reference fingerprints had been recorded, several correlation databases were constructed.

**Figure 11:**
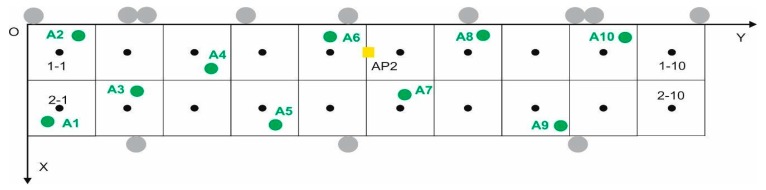
Distribution of the 10 random points or target points.

In the online phase, the same laptop used in the offline phase was used to record between 10 and 26 target points (depending on the test) randomly in the grid (some are shown in Figure 11 as green points), 30 one-second samples every 2 s (15 samples) and for each orientation (N, E, S, W), their coordinates also being recorded with the laser pointer. The structure for each of the target fingerprints is: (xo,yo,T) where (xo,yo) are the coordinates of each random point and T has the following structure:

(5) $$T=\left[\begin{array}{ccc} id_{j} & or_{k} & rss_{jk}\\ \vdots & \vdots & \vdots \end{array}\right]$$

##### Test 1. Mean Values by Orientation 

From the original database, another database was built, which we shall call Wi-Fi_0, in which starting from the $R_{i}$ values the mean RSS values for each orientation were obtained, having been reduced to 240 entries (20 points × 4 orientations × 3 APs). With 10 target points, three samples were randomly selected from each AP and orientation at consecutive time points (6 s) and their mean value calculated. From there, the distance (in signal space) between each reference fingerprint for the four orientations and each target fingerprint (by orientation) was calculated, i.e., each north-facing target fingerprint was compared with all the reference fingerprints for the four orientations, etc.

The purpose of this first test was to simulate the positioning of a user with a mobile device who is specifically facing in one of the four possible directions in real time. These data could be ascertained through the device compass. The accuracy results are shown in Table 2 (for the first 4 *k*-values) and the precision results in Table 3.

It may be seen from the above data that accuracy and precision are clearly superior when facing West, with an average accuracy for *k* = 4 neighbours greater than 4 m and a precision of less than 5.21 m 90% of the time. The worst-performing orientations were north and south. One possible explanation for this is that on the north-south axis, the person who records the data used to build the reference database screens the signal from the three APs to a certain extent, as shown in Figure 7 in the preliminary analysis.

##### Test 2 and 3: Mean Values by Orientation 

In the offline phase and to eliminate the orientation effect we followed an approach similar to [4]: from the original database we obtained the mean RSS values for each orientation and subsequently selected the maximum value from the four orientations, reducing it to 60 entries (20 points × 3 AP). We shall call this database Wi-Fi_1. In the online phase for 26 target points the following tests were carried out:

(a) Test 2. The same procedure was followed as with the reference points: the mean values of all the RSS samples were obtained for each orientation and AP, and subsequently the maximum value from the 4 orientations was selected. This approach is not real since a mobile device user would never stop to take 15 samples in 30 s facing the four cardinal directions. Nevertheless, it will serve a purpose as a method of comparison for the following approach, which we consider closer to reality.
(b) Test 3. Nine samples were randomly taken: three consecutive samples for each AP in the same time interval and in a specific orientation, this orientation varying between the different target points. The maximum value was then calculated. From there, the distance (in signal space) between each reference fingerprint and each target fingerprint was calculated.

**Figure 12:**
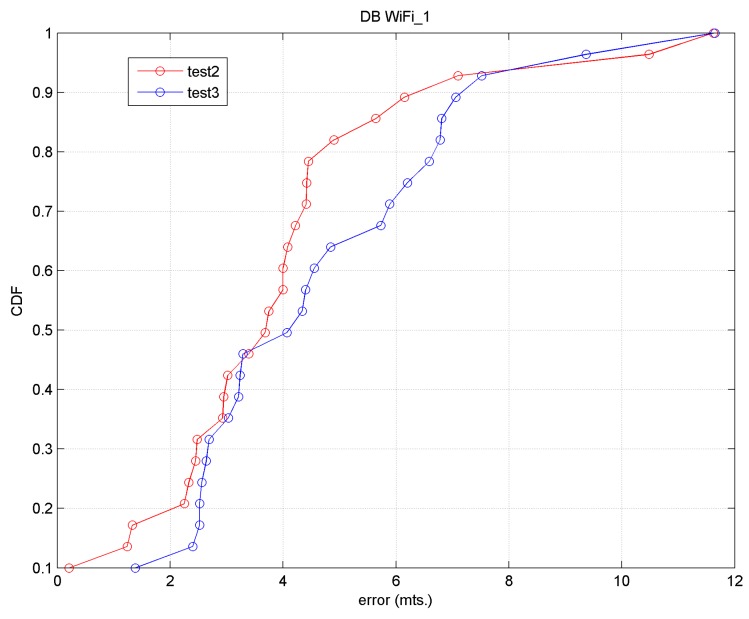
Precision (CDF) for Test 2 and Test 3.

The accuracy results for both tests are shown in Table 4 (for the first 4 *k*-values) and the precision results using the CDF in Figure 12.

In general we observed that the approach in Test 2 produced a greater accuracy than that of Test 3; the precision of Test 2 is less than 4 m 65% of the time, and the precision of both tests is less than 8 m approximately 92% of the time. Therefore, accuracy and precision values are obtained from the order of the desired cell or the cell immediately adjacent.

We must keep in mind that: (1) we are working with raw data; (2) we are working in an environment with considerable noise produced by the continuous movement of passengers and buses; (3) we have chosen a grid with cells spaced every 4 m and few reference points, precisely because we want to adjust Wi-Fi positioning with BLE positioning.

The focus of Test 3 is closer to reality and it was observed that a user would be located in a cell immediately next to the real cell in 50% of cases.

##### Test 4. Spearman’s Rank Correlation Coefficient 

In this test, the Wi-Fi_1 database is taken and the RSS values are ranked in order to then apply Spearman’s rank correlation coefficient [29,32]. This index ρ , is used primarily to calculate the correlation between the reference fingerprints and the target fingerprints:

ρ={(6a)1−6·∑i=1N(ti−ri)2N·(N2−1)     (6b)∑i=1N(Vt(i,2)−ρ¯t)·(Vr(i,2)−ρ¯r)∑i=1N[Vt(i,2)−ρ¯t]2·∑i=1N[Vr(i,2)−ρ¯r]2     

where ${\bar{\rho }}_{t,r}=\frac{1}{N}\cdot \overset{N}{\underset{i=1}{\sum }}V_{t,r}\left(i,2\right)$ .

Equations (6a) or (6b) are used if data do not, or do, have tied ranks. $V_{t}$ , $V_{r}$ are two Nx2 matrices that are built to account for the fact that target and reference fingerprints might not have the same number of APs, nor the same APs. 

In this case, the four mean orientations of the 3 APs (for each reference fingerprint, R, and target fingerprint, T) are taken as if they were distinct APs, i.e., as if there were 12 APs, and the correlation coefficient is calculated between both fingerprints, ρ , with −1≤ρ≤1 . A value of ρ=−1 indicates that there is no correlation between the fingerprints and a value of 1 indicates maximum correlation. Using this index, it is possible to calculate the distance between fingerprints in the form d=1−ρ , the values of which will be in the range 0–2.

**Figure 13:**
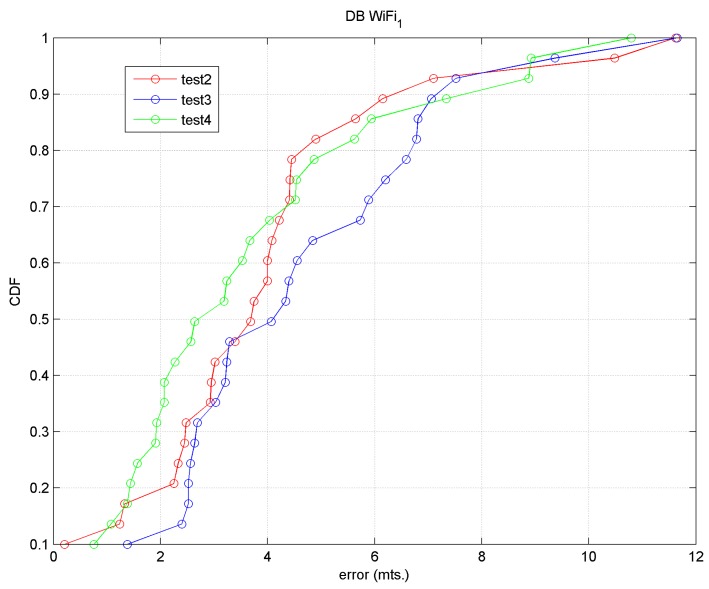
Precision (CDF) for Test 2, Test 3 and Test 4.

In this test, 26 target points were again taken; the accuracy results are displayed in Table 5, compared with the results of Tests 2 and Test 3. Figure 13 shows the precision results in relation to the results of Test 2 and Test 3.

We may observe that for *k* = 2 with Test 4, a mean error of 3.94 m and a precision of 3.19 m 50% of the time and near to 8.0 m 90% of the time are obtained.

### 5.2. BLE Analysis

#### 5.2.1. Preliminary Analysis

As may be seen in Figure 1 and Figure 4, the distribution of the seven chosen beacons was as follows: four beacons (B1, B3, B5, B7) in the boarding area and at alternate stops and three beacons (B2, B4, B6) in the area separate from the boarding area (8 m away). The reason for choosing this distribution, which is not ideal (the ideal would be a beacon at each stop), was to achieve better BLE positioning in areas away from the boarding points. As was the case with Wi-Fi, certain RSS properties were also studied in the testbed and under the same conditions.

**Figure 14:**
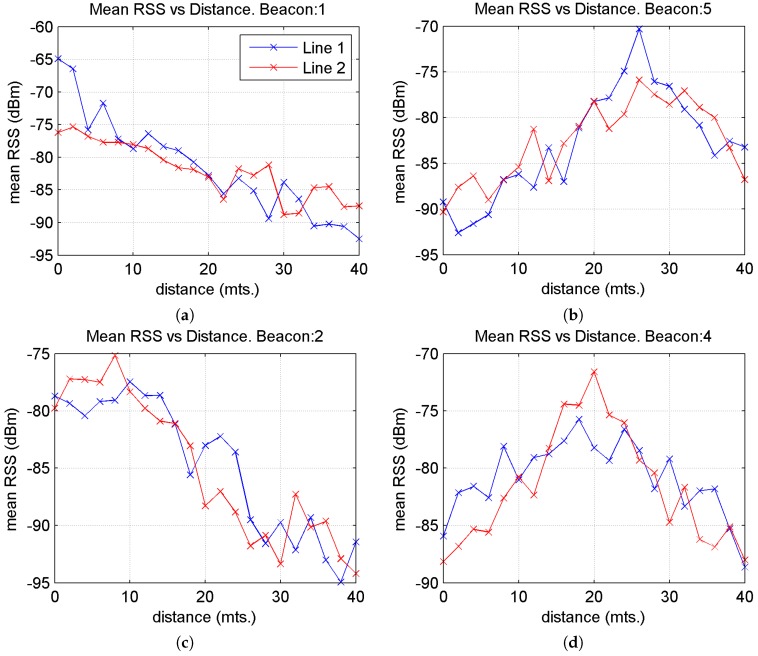
Graphs for the mean RSS values vs. distance to the Y axis at reference points along lines 1 and 2, for 4 of the 7 beacons. (**a**) Beacons 1; (**b**) Beacons 5; (**c**) Beacons 2 and (**d**) Beacons 4.

Initially we studied the variation of the mean RSS values depending on the distance when moving along reference point lines 1 and 2 and for different beacons. Figure 14 displays graphs for four beacons: beacons 1 and 5 along line 1 and beacons 2 and 4 along line 2. These beacons were chosen by way of example since the behaviour is similar for all the other beacons.

The expected behaviour of this variation was observed: a decrease in mean RSS values as the distance from the beacon increases or vice versa, in addition to fluctuations due to the different behaviours of the three BLE channels. For example, in the first of the graphs, as the user moves along reference point line 1 along a north-south axis (which progressively increases the distance from beacon 1) the mean RSS value decreases. In the case of the second graph, for beacon 5, the mean RSS value increases, attaining a maximum value (when at the same Y-coordinate), then decreases, as the user moves away from the beacon towards the southern zone of the testbed.

Based on these observations we may construct an RSS attenuation model for BLE as defined by the following Equation [29]:

(7) $$P\left(d\right)=P\left(d_{0}\right)-10n\cdot {log}_{10}\left(\frac{d}{d_{0}}\right)$$

where P(d) is the RSS value at a distance of d metres from the beacon, $d_{0}$ is a reference distance, and n is the slope. If we take $d_{0}=1$ , the equation is expressed in the following form:

(8) $$P\left(d\right)=A-10n\cdot {log}_{10}\left(d\right)$$

where A is the RSS value at the reference distance. 

**Figure 15:**
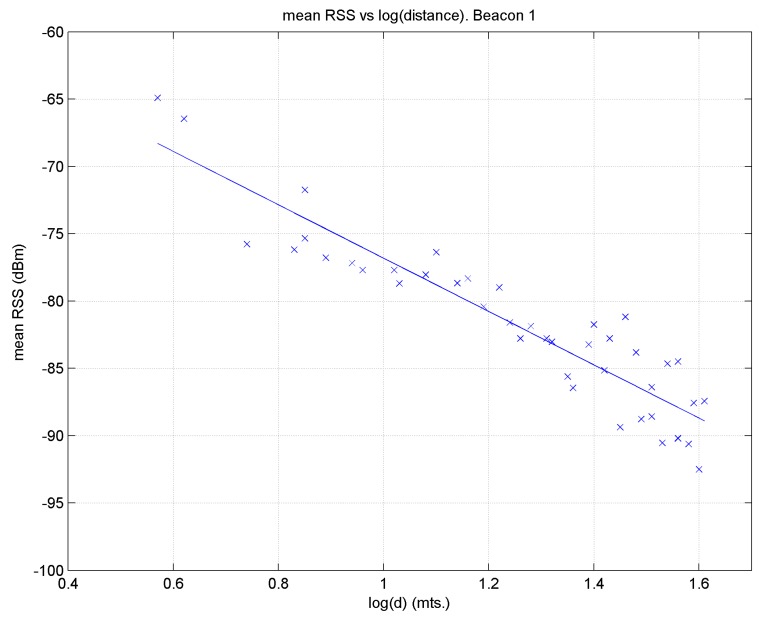
Linear regression for beacon 1 using all the reference points.

The specific process would be as follows: in the offline phase and at each reference point, the mean RSS values for each beacon are taken and the distance from the point to the corresponding beacon is calculated. Subsequently a linear regression is used to estimate the parameters A and n . Figure 15 displays the regression line for beacon 1.

One drawback of this method is that a regression line with a single slope may not correctly reflect the propagation characteristics near or far from a beacon, as these characteristics may be very different in the two zones; this would lead to sizeable errors when estimating distance, especially at remote target points. This behaviour may be observed in Figure 15 for distance logarithm values between approximately 1.4 and 1.6. To fit the model better, two regression lines could be used; for example, in relation to Figure 15, one for points to the left of the break-point (which in this case would be close to 1.4) and another for the rest, where this point would be determined empirically [29,33].

**Figure 16:**
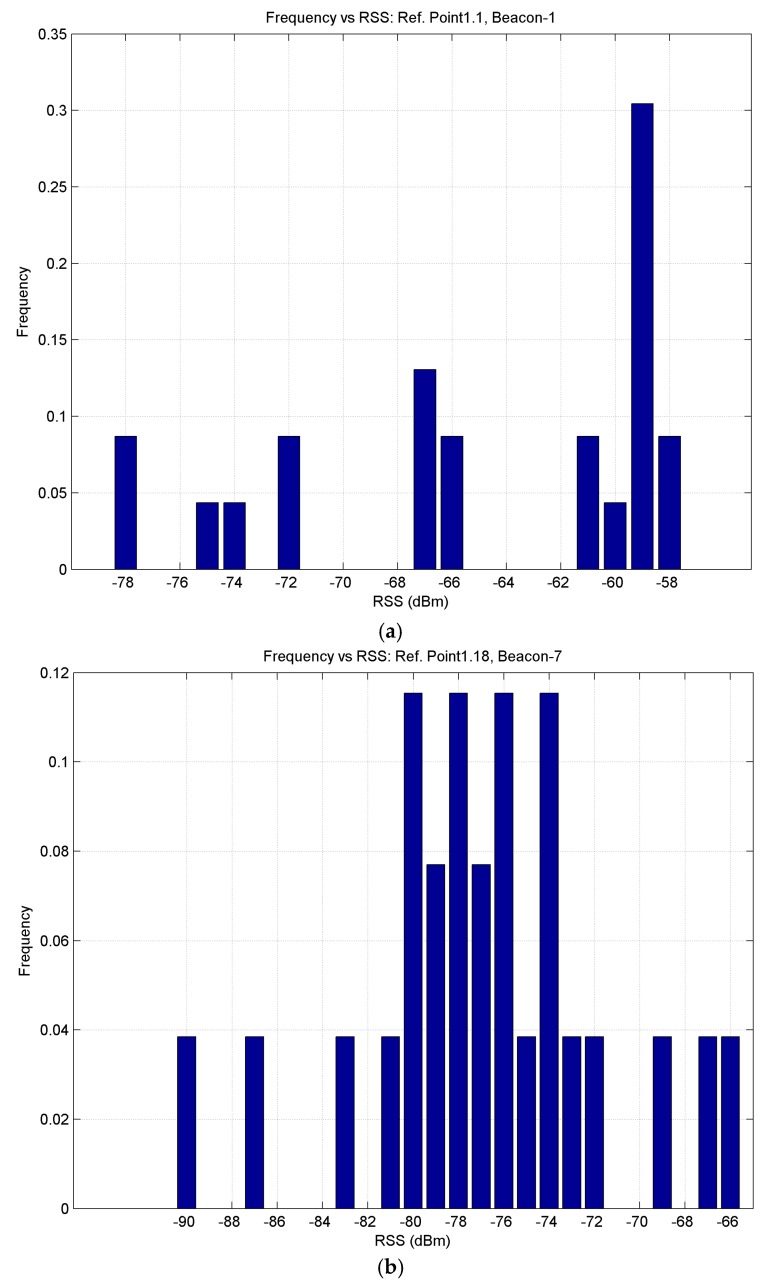
Histograms of RSS values for two beacons: (**a**) Beacon 1 in the absence of passengers; (**b**) Beacon 7 in the presence of passengers.

An initial study was also carried out to analyse the structure of the histograms for different beacons recorded by an observer located statically at several reference points. Measurements were taken for 1 min and at a distance of 1.4 m from several beacons, in the absence and presence of moving passengers. In the absence of moving passengers, the histograms presented characteristics as displayed in Figure 16a: one dominant RSS value was recorded (in this case, 59 dBm). However, in the presence of moving passengers, the histograms presented characteristics as displayed in Figure 16b: there is no longer a dominant value.

At the same distance from a beacon, the observed RSS value is not the same for the three BLE channels (37, 38 and 39). In our case, even working in aggregate mode, this effect is reflected in the multimodal distribution shown in the histogram of Figure 16b; this is simply the consequence of the fact that BLE technology transmits advertisements on these channels to mitigate interference with other devices [15].

#### 5.2.2. Empirical Approach to the BLE Tests

The empirical approach followed was similar to that followed with Wi-Fi [34]. In this case, however, a grid of 22 cells, each measuring 2 m × 2 m with a density of 14.5 fingerprints per m^2^, was chosen initially (later increased to 42 cells), and seven BLE beacons were deployed (see Figure 5). For these tests, the configuration of each beacon was not very cost-effective in terms of battery consumption: at a frequency of 50 Hz and with an output power of 0 dBm.

We should note that the beacons are located on alternate columns, which means a beacon at alternate bus stops. The density of beacons is not ideally suited to the purposes of our study (to position a user at the desired stop and offer advanced services) but it may give us an idea of a higher level of positioning error for this technology and in this environment.

In the offline phase, with a Samsung Galaxy S5 mobile device running Android 6.0.1 (Samsung Electronics, Seoul, Korea) and the Beacon Scanner application, 1 sample per second was taken at each reference point for 30 s and for each beacon. The scanning was performed in aggregate mode, i.e., without individually distinguishing BLE channels 37, 38 and 39. Therefore, the initial and final database of reference points consisted of approximately 4620 entries (22 points × 30 samples × 7 beacons) and 8820 entries (42 points × 30 samples × 7 beacons). Once the raw reference fingerprints had been recorded, several correlation databases were constructed.

##### Test 1, Test 2 and Test 3. Mean and Median Values

**Figure 17:**
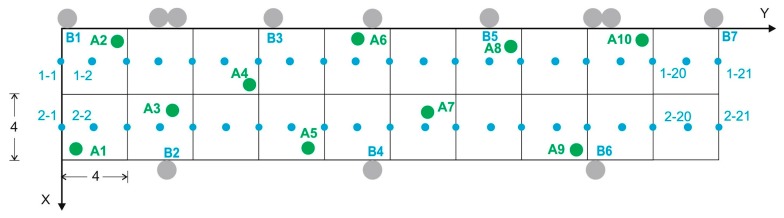
Grid with the initial 22 reference points (only odd points) and with the 42 points used later. The 10 target points are also displayed.

From the original database three databases were created, which we shall call BLE_1, BLE_2 and BLE_3, in which the mean RSS values (BLE_1 and BLE_3) and the median RSS values (BLE_2) were obtained for each beacon, said databases having been reduced to 154 (22 points × 7 beacons) and 294 entries each (42 points × 7 beacons) depending on the number of reference points (22 or 42). In the online phase, 10 target points were used, the same points that had been used for Wi-Fi positioning (see Figure 17). With the same mobile device used in the offline phase, a procedure was carried out for the databases similar to the reference fingerprints procedure.

**Figure 18:**
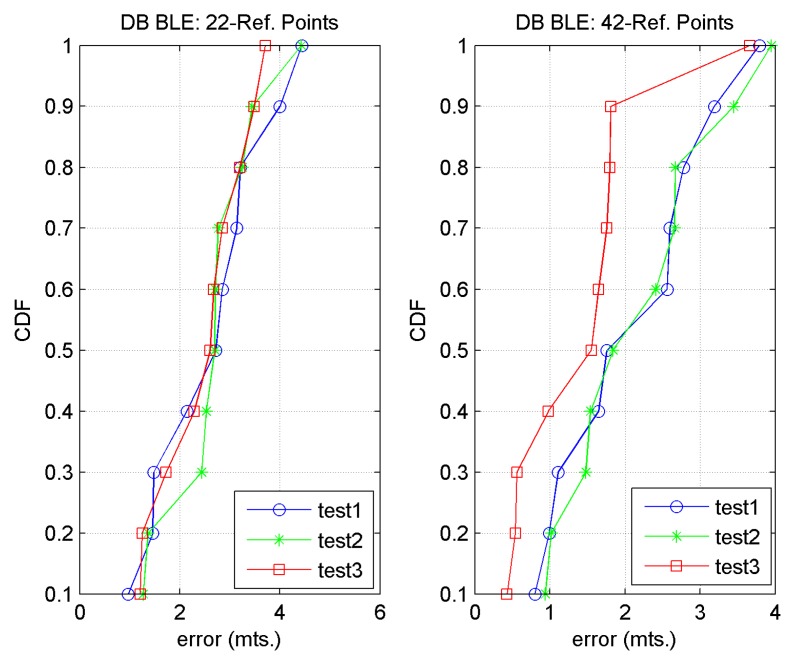
Precision (CDF) for Test 1, 2 and 3 with: (**a**) 22 and (**b**) 42 reference points.

From there, the distance (in signal space) between each reference fingerprint and each target fingerprint was calculated using two distance metrics: (a) Euclidean (BLE_1 and BLE_2), and (b) Mahalanobis (BL_3) [10], where the fingerprint comparison implemented in the three cases was the WKNN algorithm. The accuracy results of the three tests are shown in Table 6 (for the first 4 *k*-values) and the precision results in Table 7, Table 8 and Figure 18.

We can see that using 42 reference points, better values of accuracy and precision are obtained. In this case, the accuracy of Tests 1 and 2 is very similar, although it is slightly higher for Test 1, where mean RSS values were used. The Test with the best results is Test 3 (mean RSS values and Mahalanobis distance), which obtained a mean error of 1.47 m for *k* = 2 neighbours. With regard to precision, for *k* = 2 the best behaviour is that of Test 3, which obtained an error of 1.81 m 90% of the time. The above data show that, using the Mahalanobis distance, the mean RSS value, and for *k* = 2 neighbours, we obtained an average accuracy of 1.47 m and a precision of approximately 1.81 m 90% of the time.

#### 5.2.3. RSS Attenuation Model for BLE

The method for obtaining the regression line seen in Section 5.2.1 can be used to calculate the distances of the target points from each beacon, which would mean knowing the distance from each target point to each bus stop. The process would be as follows: ascertain the mean RSS value of a target point, *T*, and isolate d from the equation of the regression line (obtained for reference points), to then obtain an estimate of the distance from point *T* to a specific beacon. For example, for 42 reference points and beacon 1, the regression line obtained was:

(9) $$P\left(d\right)\;=\;-57.0-19.8\cdot \log_{10}\left(d\right)$$

Isolating d from these equations and knowing the mean RSS values at the target points, we obtain the estimated distance to the corresponding beacon (B1 of Figure 5b in this case). The actual distance is obtained by calculating Euclidean distance between the target point and beacon 1.

If two regression lines are used, the following equations are obtained:

(10) $$P\left(d\right)\;=\;\{\begin{array}{c} -57.4-19.4\cdot \log_{10}\left(d\right),\;\;\;\;\;{\;\log}_{10}\left(d\right)\leq 1.31\\ -53.7-22.0\cdot log_{10}\left(d\right),\;\;\;\;\;{\;\log}_{10}\left(d\right)>1.31 \end{array}$$

**Figure 19:**
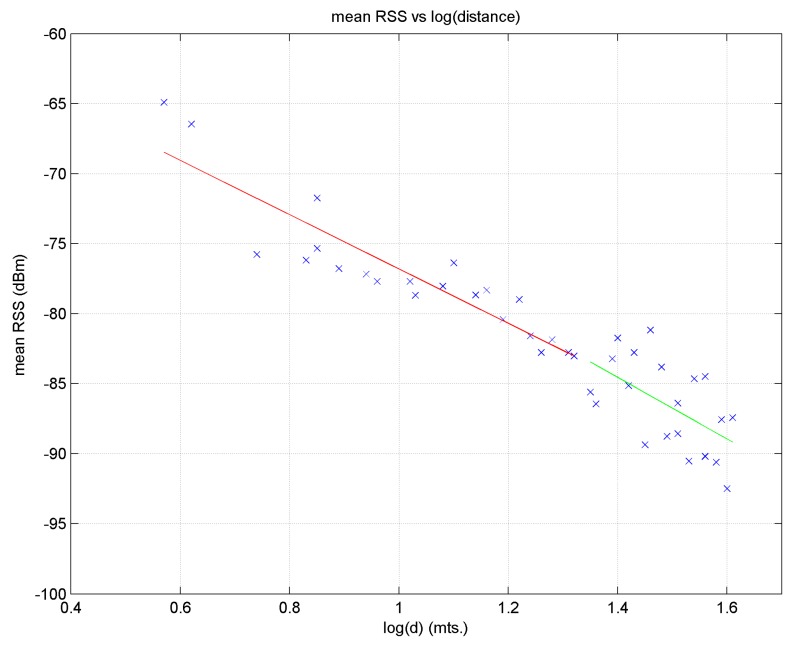
Linear regression for beacon 1 using all the reference points.

The graph in Figure 19 shows the regression line for beacon 1 using line Equation (10).

Table 9 shows the estimation of the distance of 10 target points from beacon 1 using regression line (3) and regression lines (4), compared with the actual distance.

If one regression line is used, the mean error in the distance estimate for beacon 1 is 1.91 m and if two lines are used, the mean error falls to 1.85 m; the error in both cases is less than 2.0 m 70% of the time.

## 6. Discussion

It is not a simple task to compare our proposed system with other studies. The main reason for this is that each system has a range of parameters (number of access points/beacons, location of said access points, ambient conditions and working environment, etc.) which are very different in each study. If we add to this the fact that not all authors express accuracy and precision in the same way, it becomes evident that it is very difficult to establish a complete comparison between systems. Despite this fact, a comparison between the results of our study and the results obtained by other systems is presented below.

In Table 10, accuracy and precision for some wireless-based indoor positioning systems are shown. For WLAN-based systems, accuracy values are in the range of 1.5–5.0 m, and precision values in the range of 2.0–5.9 m 90% of the time. With respect to Bluetooth-based systems, accuracy values are in the range of 0.48–3.6 m and precision values in the range of 2.0–4.8 m 95% of the time.

Table 11 and Table 12 show accuracy and precision for the different tests presented in this paper. WLAN accuracy is in the range of 3.94–4.82 m, and precision in the range of 5.21–7.0 m 90% of the time. With respect to BLE-based tests, accuracy is in the range of 1.47–2.15 m, and precision in the range of 1.81–3.58 m 90% of the time.

Comparing results, it can be observed that for WLAN, with only three APs, the accuracy values obtained in this paper are in the same range as the values obtained by the systems shown in Table 10, while precision results are slightly out of this range, especially the upper limit. With respect to BLE, with a density of one beacon per 45.7 m^2^, the values of accuracy and precision obtained in this study are in the same range. Taking into account the fact that the usual values of accuracy and precision obtained in laboratory conditions are similar to our results, we consider that our results, even when obtained in challenging conditions, are reasonable.

## 7. Conclusions and Future Works

In this paper, we have presented a positioning system based on two subsystems: Wi-Fi and Bluetooth Low Energy. The first (Wi-Fi) was intended to position users with not very high levels of accuracy and precision, but not too far from reality; the second (BLE) was intended to position the user with greater precision, as well as to provide advanced information services at the destination point. For both types of positioning, preliminary analyses were carried out of the behaviour of the received signal strength, the suitability of the measuring devices, etc. For Wi-Fi positioning, four tests were carried out, and for Bluetooth, three tests along with a signal attenuation model. For all the tests, the Euclidean distance metric was used, due to its simplicity of implementation and acceptable results, except for one of the BLE tests in which the Mahalanobis distance was used; the fingerprint comparison algorithm applied was WKNN.

In all the Wi-Fi tests, measurements were taken at reference points spaced every 4 m. The first took into account the orientation of the user, the online phase of which described a scenario very close to reality as it took only three samples of the signal from each access point. Two orientations presented better results than the other two, probably due to the arrangement of the access points with respect to the person measuring the data, who screens the signal coming from these access points. For the most favourable orientation, and compared with four neighbours, an average accuracy of 4.44 m and a precision of less than 5.21 m 90% of the time were obtained. In the second and third tests, the user orientation effect was eliminated, producing, respectively, an accuracy of 4.13 and 4.82 m compared to two neighbours. The precision of the two tests was less than 4.0 m 50% and 60% of the time, respectively. In the fourth and final test, the mean orientations were taken and Spearman’s rank correlation coefficient was used to compare reference and target fingerprints, obtaining by comparison with two neighbours an accuracy of 3.94 m and a precision of less than 4 m almost 70% of the time.

With regard to the three BLE tests, measurements were taken at reference points spaced every 1 and 2 m, obtaining better results in the first case. The data collection was carried out in aggregate mode, i.e., without differentiating between channels 37, 38 and 39. The primary difference between the first two tests was that in the first the mean RSS value was taken and in the second, the median. In both tests very similar accuracy results were obtained by comparison with two neighbours, with position values very close to 2.0 m. Regarding precision, the first test behaved better with three neighbours, obtaining a precision of less than 2.51 m 90% of the time. The third test, which used mean RSS values and Mahalanobis distance, gave the best results: for *k* = 2 neighbours, a mean error of 1.47 m and a precision of 1.81 m was obtained 90% of the time. In the case of the attenuation model, we compared results by fitting the data to one or two regression lines. An example was presented in which if one regression line is used, the mean error in the distance estimate from a specific beacon is 1.91 m and, if two lines are used, the mean error falls to 1.85 m, with the error in both cases being less than 2.0 m 70% of the time. 

Taking into account the fact that this system is designed to work in real situations in a scenario with high environmental fluctuations, and comparing those results with others obtained in laboratory scenarios, our results are promising and demonstrate that the system would be able to position users with these reasonable values of accuracy and precision. That would place a user in the desired cell, or in the immediately adjacent cell, in the worst case, on a high percentage of occasions, as was originally required. In addition, we can conclude that for BLE positioning, excellent accuracy and precision values are achieved, so this method would position a user with even greater precision than Wi-Fi positioning.

We understand that, for the conditions in which this system was deployed, the error values obtained for both positioning systems, but mainly for Wi-Fi, reflect upper bounds and could be improved, making this system a strong candidate for deployment in other similar environments. To this end, and as future lines of action, we may mention the following: for its low cost and ease of maintenance, a greater deployment of BLE transmitters, use of grids with reference points with smaller spacing, data collection campaigns with greater numbers of samples, reduction of their duration, e.g., by involving (explicitly or implicitly) users or other methods, pre-processing of data and search filtering in the reference point database, use of a greater number of heterogeneous devices (with corresponding calibration) for the offline and online data collection phases, studies of probabilistic positioning algorithms and comparison of the results obtained with other distance metrics that may be suitable for this type of environment.

## Figures and Tables

**Table 1:** Zones of the testbed with the same RSS order.

**Line 1**	1-1	1-2	1-3	1-4	1-5	1-6	1-7	1-8	1-9	1-10
**RSS order**	123	213	213	213	231	231	231	231	231	321
**Line 2**	2-1	2-2	2-3	2-4	2-5	2-6	2-7	2-8	2-9	2-10
**RSS order**	213	213	231	213	231	231	231	231	231	321

**Table 2:** Accuracy of Test 1: mean errors for different *k* and orientation values.

Orient.	*k* = 1	*k* = 2	*k* = 3	*k* = 4
**N**	5.83	6.68	6.97	7.39
**S**	7.08	6.95	7.47	6.81
**E**	8.10	7.05	7.46	7.63
**W**	5.25	5.34	4.54	4.44

**Table 3:** Precision of Test 1.

**Perc**	***k* = 1**				***k* = 2**			
	**N**	**E**	**S**	**W**	**N**	**E**	**S**	**W**
**25%**	3.21	3.15	5.53	1.73	3.68	2.54	4.75	2.97
**50%**	5.05	5.53	7.37	4.72	5.05	4.75	6.18	4.75
**90%**	9.24	11.38	13.44	8.12	9.24	11.47	11.47	8.39
**Perc**	***k* = 3**				***k* = 4**			
	**N**	**E**	**S**	**W**	**N**	**E**	**S**	**W**
**25%**	3.75	3.71	4.36	3.57	4.66	3.71	4.53	3.13
**50%**	4.99	4.53	5.52	3.67	5.80	4.53	8.48	3.88
**90%**	14.12	9.43	10.08	6.73	13.88	8.52	9.43	5.21

**Table 4:** Comparison of the accuracy of Test 2 and Test 3 for the Wi-Fi_1 database: mean errors for different *k*-values.

	*k* = 1	*k* = 2	*k* = 3	*k* = 4
**Mean error Test 2**	5.00	4.13	4.15	4.49
**Mean error Test 3**	6.14	4.82	5.52	5.41

**Table 5:** Comparison of the accuracy of Test 2, Test 3 and Test 4 for the Wi-Fi_1 database: mean errors for different *k*-values.

	k = 1	k = 2	k = 3	k = 4
Mean error Test 2	5.00	4.13	4.15	4.49
Mean error Test 3	6.14	4.82	5.52	5.41
Mean error Test 4	4.61	3.94	4.25	3.97

**Table 6:** Accuracy of Tests 1, 2 and 3: mean errors for different *k*-values.

*k*	22 Reference Points			42 Reference Points		
	Mean Error (Test 1:BLE_1)	Mean Error (Test 2:BLE_2)	Mean Error (Test 3:BLE_3)	Mean Error (Test 1:BLE_1)	Mean Error (Test 2:BLE_2)	Mean Error (Test 3:BLE_3)
**1**	2.96	3.03	2.70	2.10	2.11	1.86
**2**	2.64	2.69	2.50	2.02	2.12	1.47
**3**	2.67	2.72	2.65	2.02	2.15	2.05
**4**	2.44	2.58	2.96	2.36	2.34	2.09

**Table 7:** Precision of Tests 1, 2 and 3. 22 reference points.

Perc	Test 1:BLE_1				Test 2:BLE_2				Test 3:BLE_3			
	*k* = 1	*k* = 2	*k* = 3	*k* = 4	*k* = 1	*k* = 2	*k* = 3	*k* = 4	*k* = 1	*k* = 2	*k* = 3	*k* = 4
**25%**	1.15	1.48	2.37	2.16	1.15	2.44	2.29	2.23	1.17	1.73	2.33	2.29
**50%**	1.55	2.72	2.88	2.49	2.14	2.70	2.64	2.55	2.14	2.61	2.57	2.84
**90%**	5.12	4.00	3.10	3.31	5.12	3.45	3.58	3.66	4.60	3.49	3.33	3.83

**Table 8:** Precision of Tests 1, 2 and 3. 42 reference points.

Perc	Test 1:BLE_1				Test 2:BLE_2				Test 3:BLE_3			
	*k* = 1	*k* = 2	*k* = 3	*k* = 4	*k* = 1	*k* = 2	*k* = 3	*k* = 4	*k* = 1	*k* = 2	*k* = 3	*k* = 4
**25%**	1.09	1.11	1.26	1.80	1.09	1.48	1.19	1.82	1.09	0.56	1.64	1.70
**50%**	1.65	1.65	2.21	2.20	1.65	1.68	2.09	1.95	1.65	1.55	2.13	1.96
**90%**	3.37	3.19	2.51	3.29	3.37	3.45	3.24	3.12	2.79	1.81	2.43	2.67

**Table 9:** Estimation of distances of 10 target points from beacon 1 by means of the signal attenuation model with 1 and 2 regression lines.

Beacon 1										
**RL 1 SLOPE**	**T1**	**T2**	**T3**	**T4**	**T5**	**T6**	**T7**	**T8**	**T9**	**T10**
Mean RSS (dBm)	−77.5	−70.1	−75.0	−78.4	−81.0	−81.3	−85.6	−85.6	−86.5	−88.7
Actual distance (m)	7.28	4.11	8.76	11.93	17.04	18.82	23.35	27.07	32.61	35.48
Estimated distance (m)	10.83	4.60	8.12	12.04	16.39	17.00	28.05	28.04	31.11	40.11
**Error (m)**	**3.55**	**0.49**	**0.63**	**0.12**	**0.65**	**1.82**	**4.70**	**0.97**	**1.49**	**4.63**
**RRL 2 SLOPES**	**T1**	**T2**	**T3**	**T4**	**T5**	**T6**	**T7**	**T8**	**T9**	**T10**
Mean RSS (dBm)	−77.5	−70.1	−75.0	−78.4	−81.0	−81.3	−85.6	−85.6	−86.5	−88.7
Actual distance (m)	6.92	4.11	8.76	11.93	17.04	18.82	23.35	27.07	32.61	35.48
Estimated distance (m)	10.85	4.53	8.09	12.10	16.58	17.20	28.28	28.27	31.04	39.02
**Error (m)**	**3.93**	**0.41**	**0.67**	**0.17**	**0.47**	**1.62**	**4.93**	**1.20**	**1.56**	**3.54**

**Table 10:** Some wireless-based indoor positioning systems, studies and solutions [5,35,36].

System/Study/Solution	Wireless Technologies	Positioning Algorithm	Accuracy	Precision
RADAR	WLAN RSS	KNN	3–5 m	50% within around 2.5 m and 90% within around 5.9 m
Horus	WLAN RSS	Probabilistic	2 m	90% within 2.1 m
DIT	WLAN RSS	MLP, SVM	3 m	90% within 5.12 for SVM; 90% within 5.40 for MLP
Ekahau	WLAN RSSI	Probabilistic	2–3 m	50% within 2 m
MultiLoc	WLAN RSS	SMP	2.7 m	50% within 5.4 m
Faragher [15]	BLE	Bayesian approach	N/A	95% within 2.6 m (1 beacon per 30 m^2^)–4.8 m (1 beacon per 100 m^2^)
Zhu [33]	BLE	RSSI propagation model; Gaussian filter; Piecewise fitting for offline training; Weighted distance filter	N/A	80% within 1.5 m
Cinefra	BLE	Path loss model with particle filter to estimate parameters	0.48–2.06 (office env.); 0.66–3.6 (home env.)	50% within 1.5 m
Indoo.rs	BLE	Fingerprinting indoor positioning approach	N/A	95% within 5.0 m
Zhuang [16]	BLE	channel-separate polynomial regression model, channel-separate fingerprinting, outlier detection and extended Kalman filtering	N/A	90% within 2.56 m (1 beacon per 9 m^2^)–3.88 m (1 beacon per 18 m^2^)

**Table 11:** Summary of WLAN tests presented in this study.

Test	Method	Accuracy	Precision
Test 1	Offline: mean RSS for orientation. Online: 3 consecutive samples. Euclidean distance + WKNN	4.44 m	90% within 5.21 m
Test 2	Offline + online: max. Value of mean RSS for orientation. Euclidean distance + WKNN	4.13 m	90% around 6 m
Test 3	Offline: max. Value of mean RSS for orientation. Online: 3 consecutive samples in a specific orientation. Euclidean distance + WKNN	4.82 m	90% around 7 m
Test 4	Offline + online: ranked mean RSS for orientation. Spearman’s rank + WKNN	3.94 m	90% around 7 m

**Table 12:** Summary of BLE tests presented in this study.

Test	Method	Accuracy	Precision
Test 1	Offline + online: mean RSS. Euclidean distance +WKNN	2.02 m	90% within 3.10 m
Test 2	Offline + online: median RSS. Euclidean distance +WKNN	2.15 m	90% within 3.58 m
Test 3	Offline + online: mean RSS. Mahalanobis distance +WKNN	1.47 m	90% within 1.81 m

## References

[1] Good Practices of Accessible Urban Development. http://www.un.org/disabilities/documents/desa/good_practices_in_accessible_urban_development_october2016.pdf.

[2] European Conference of Ministers of Transport (2006). Improving Transport Accessibility for All. Guide to Good Practice.

[3] Mitchell C.G.B., Suen S.L. (1998). Urban Travel, Intelligent Transportation Systems, and the Safety of Elderly and Disabled Travelers. J. Urban Technol..

[4] Bahl P., Padmanabhan V.N. RADAR: An In-building RF-Based User Location and Tracking System. Proceedings of the IEEE 9th Annual Joint Conference of the IEEE Computer and Communications Societies.

[5] Liu H., Darabi H., Banerjee P., Liu J. (2007). Survey of Wireless Indoor Positioning Techniques and Systems. IEEE Trans. Syst. Man Cybern. Part C.

[6] Honkavirta V., Perälä T., All-Löytty S., Piché R. A Comparative Survey of WLAN Location Fingerprinting Methods. Proceedings of the 6th Workshop on Positioning, Navigation and Communication.

[7] He S., Chan S.H.G. (2016). Wi-Fi Fingerprint-Based Indoor Positioning: Recent Advances and Comparisons. IEEE Commun. Surv. Tutor..

[8] Kaemarungsi K., Krishnamurthy P. Properties of Indoor Received Signal Strength for WLAN Location Fingerprinting. Proceedings of the First Annual International Conference on Mobile and Ubiquitous Systems: Networking and Services.

[9] Dawes B., Chin K.W. (2011). A Comparison of Deterministic and Probabilistic Methods for Indoor Localization. J. Syst. Softw..

[10] Torres-Sospedra J., Montoliu R., Trilles S., Belmonte O., Huerta J. (2015). Comprehensive Analysis of Distance and Similarity Measures for Wi-Fi Fingerprinting Indoor Positioning Systems. Expert Syst. Appl..

[11] Feng C., Au W.S.A., Valaee S., Tan Z. (2012). Received-Signal-Strength-Based Indoor Positioning Using Compressive Sensing. IEEE Trans. Mob. Comput..

[12] Kjærgaard M.B. (2011). Indoor Location Fingerprinting with Heterogeneous Clients. Pervasive Mob. Comput..

[13] King T., Haenselmann T., Effelsberg W. (2007). Deployment, Calibration, and Measurement Factors for Position Errors in 802.11-based Indoor Positioning Systems. Lect. Notes Comput. Sci..

[14] Faragher R., Harle R. An Analysis of the Accuracy of Bluetooth Low Energy for Indoor Positioning Applications. Proceedings of the 27th International Technical Meeting of The Satellite Division of the Institute of Navigation.

[15] Faragher R., Harle R. (2015). Location Fingerprinting with Bluetooth Low Energy Beacons. IEEE J. Sel. Areas Commun..

[16] Zhuang Y., Yang J., Li J., Qi L., El-Sheimy N. (2016). Smartphone-Based Indoor Localization with Bluetooth Low Energy Beacons. Sensors.

[17] Kajioka S., Mori T., Uchiya T., Takumi I., Matsuo H. Experiment of Indoor Position Presumption Based on RSSI of Bluetooth LE Beacon. Proceedings of the IEEE 3rd Global Conference on Consumer Electronics.

[18] Baniukevic A., Jensen C.S., Lu H. Hybrid Indoor Positioning with Wi-Fi and Bluetooth: Architecture and Performance. Proceedings of the IEEE 14th International Conference on Mobile Data Management.

[19] Metola-Moreno E., Aparicio S., Tarrío-Alonso P., Casar-Corredera J.R. Comparison of Localization Methods Using Calibrated and Simulated Fingerprints for Indoor Systems Based on Bluetooth and WLAN Technologies. Proceedings of the 3rd International Workshop on User-Centric Technologies and Applications.

[20] Au A.W.S., Feng C., Valaee S., Reyes S., Sorour S., Markowitz S.N., Gold D., Gordon K., Eizenman M. (2013). Indoor Tracking and Navigation Using Received Signal Strength and Compressive Sensing on a Mobile Device. IEEE Trans. Mob. Comput..

[21] Moder T., Hafner P., Wieser M. Indoor Positioning for Visually Impaired People Based on Smartphones. Proceedings of the 14th International Conference Computers Helping People with Special Needs.

[22] Ge T. (2015). Indoor Positioning System based on Bluetooth Low Energy for Blind or Visually Impaired Users. Master’s Thesis.

[23] Guerrero L.A., Vasquez F., Ochoa S.F. (2012). An Indoor Navigation System for the Visually Impaired. Sensors.

[24] Castillo-Cara M., Huaranga-Junco E., Mondragón-Ruiz G., Salazar A., Barbosa L.O., Antúnez E.A. (2016). Ray: Smart Indoor/Outdoor Routes for the Blind Using Bluetooth 4.0 BLE. Procedia Comput. Sci..

[25] Ladd A.D., Bekris K.E., Rudys A., Kavraki L.E., Wallach D.S. (2005). Robotics-Based Location Sensing Using Wireless Ethernet. Wirel. Netw..

[26] Lin H., Zhang Y., Griss M., Landa I. (2009). WASP: An Enhanced Indoor Locationing Algorithm for a Congested Wi-Fi Environment. Lect. Notes Comput. Sci..

[27] Dickinson P., Cielniak G., Szymanezyk O., Mannion M. Indoor Positioning of Shoppers Using a Network of Bluetooth Low Energy Beacons. Proceedings of the 7th International Conference on Indoor Positioning and Indoor Navigation.

[28] Indoor Navigation for Zurich Main Railway Station. https://www.infsoft.com/industries/railway/success-story.

[29] Saraiva R., Lovisolo L. (2015). RF Positioning: Fundamentals, Applications and Tools.

[30] Youssef M., Agrawala A. The Horus WLAN Location Determination System. Proceedings of the 3rd International Conference on Mobile Systems, Applications, and Services.

[31] García-Villalonga S., Pérez-Navarro A. Influence of human absortion of Wi-Fi signal in indoor positioning with Wi-Fi fingerprinting. Proceedings of the 2015 International Conference on Indoor Positioning and Indoor Navigation (IPIN).

[32] Xie Y., Wang Y., Nallanathan A., Wang L. (2016). An Improved K-Nearest-Neighbor Indoor Localization Method Based on Spearman Distance. IEEE Signal Process. Lett..

[33] Zhang L., Liu X., Song J., Gurrin C., Zhu Z. A Comprehensive Study of Bluetooth Fingerprinting-based Algorithms for Localization. Proceedings of the 27th International Conference on Advanced Information Networking and Applications Workshops.

[34] Zhu J., Chen Z., Luo H., Li Z. RSSI Based Bluetooth Low Energy Indoor Positioning. Proceedings of the International Conference on Indoor Positioning and Indoor Navigation.

[35] Deak G., Curran K., Condell J. (2012). A survey of active and passive indoor localisation systems. Comp. Commun..

[36] Cabarkapa D., Grujic I., Pavlovic P. Comparative analysis of the Bluetooth Low-Energy indoor positioning systems. Proceedings of the 12th International Conference on Telecommunication in Modern Sattelite, Cable and Broadcasting Services.

